# The Therapeutic Potential of Carnosine as an Antidote against Drug-Induced Cardiotoxicity and Neurotoxicity: Focus on Nrf2 Pathway

**DOI:** 10.3390/molecules27144452

**Published:** 2022-07-12

**Authors:** Giuseppe Caruso, Anna Privitera, Barbara Moura Antunes, Giuseppe Lazzarino, Susan Marie Lunte, Giancarlo Aldini, Filippo Caraci

**Affiliations:** 1 Department of Drug and Health Sciences, University of Catania, 95125 Catania, Italy; annaprivitera01@gmail.com (A.P.); fcaraci@unict.it (F.C.); 2 Oasi Research Institute-IRCCS, 94018 Troina, Italy; 3 Facultad de Deportes, Universidad Autónoma de Baja California, Ensenada 22890, Mexico; barbara.moura@uabc.edu.mx; 4 Department of Biomedical and Biotechnological Sciences, University of Catania, 95123 Catania, Italy; lazzarig@unict.it; 5 Ralph N. Adams Institute for Bioanalytical Chemistry, University of Kansas, Lawrence, KS 66047, USA; slunte@ku.edu; 6 Department of Pharmaceutical Chemistry, University of Kansas, Lawrence, KS 66047, USA; 7 Department of Chemistry, University of Kansas, Lawrence, KS 66047, USA; 8 Department of Pharmaceutical Sciences, University of Milan, 20133 Milan, Italy; giancarlo.aldini@unimi.it

**Keywords:** carnosine, antidote, cardiotoxicity, neurotoxicity, nuclear factor erythroid 2–related factor 2 (Nrf2)

## Abstract

Different drug classes such as antineoplastic drugs (anthracyclines, cyclophosphamide, 5-fluorouracil, taxanes, tyrosine kinase inhibitors), antiretroviral drugs, antipsychotic, and immunosuppressant drugs are known to induce cardiotoxic and neurotoxic effects. Recent studies have demonstrated that the impairment of the nuclear factor erythroid 2–related factor 2 (Nrf2) pathway is a primary event in the pathophysiology of drug-induced cardiotoxicity and neurotoxicity. The Nrf2 pathway regulates the expression of different genes whose products are involved in antioxidant and inflammatory responses and the detoxification of toxic species. Cardiotoxic drugs, such as the anthracycline doxorubicin, or neurotoxic drugs, such as paclitaxel, suppress or impair the Nrf2 pathway, whereas the rescue of this pathway counteracts both the oxidative stress and inflammation that are related to drug-induced cardiotoxicity and neurotoxicity. Therefore Nrf2 represents a novel pharmacological target to develop new antidotes in the field of clinical toxicology. Interestingly, carnosine (β-alanyl-l-histidine), an endogenous dipeptide that is characterized by strong antioxidant, anti-inflammatory, and neuroprotective properties is able to rescue/activate the Nrf2 pathway, as demonstrated by different preclinical studies and preliminary clinical evidence. Starting from these new data, in the present review, we examined the evidence on the therapeutic potential of carnosine as an endogenous antidote that is able to rescue the Nrf2 pathway and then counteract drug-induced cardiotoxicity and neurotoxicity.

## 1. Introduction

Toxicological emergencies are often encountered in intensive care unit practice. They can be the result of a drug overdose, or due to drug toxicity that is linked to the use of inappropriate dosages and/or unexpected drug interactions [1]. Cardiotoxicity represents one of the most common adverse drug effects [2], but different drugs have also been shown to induce clinically relevant neurotoxic effects [3].

Preventing adverse drug effects represents a mandatory step in clinical toxicology, and, in this scenario, antidotes are essential in counteracting a drug’s toxicity in combination with supportive care [4]. Different definitions of antidotes have been proposed, but one of the most validated is the one that is proposed by the International Programme of Chemical Safety, according to which an antidote can be defined as a drug that can neutralize the toxicity that is induced by a xenobiotic [5]—particularly, xenobiotics with a restricted therapeutic index and a low safety profile. The therapeutic index or ratio (TD_50_/ED_50_), defining the safety level of a molecule, represents the ratio between the toxic dose (TD) (also referred to as the lethal dose (LD)) and the effective dose (ED) at a preclinical level. According to this scenario, an antidote should act as “an agent able to increase the mean toxic/lethal dose of a toxin” [6]. Different mechanisms can underlie the clinical efficacy of currently available antidotes [4], such as: (i) preventing the absorption of the toxin; (ii) directly counteracting the toxin by competitive enzyme inhibition (e.g., fomepizole) or competitive receptor blocking (e.g., naloxone); (iii) inhibiting the conversion of the toxin to more toxic metabolites (e.g., *N*-acetyl-cysteine), finally antagonizing its end organ effects and promoting the elimination of the xenobiotics [1].

An alternative mechanism that has recently been explored in the field of clinical toxicology is the activation or rescue of the endogenous protective pathways that are impaired by the toxin, such as the nuclear factor erythroid 2–related factor 2 (Nrf2) pathway [7]. Along this line, we believe that new antidotes with an interesting therapeutic potential can be examined and proposed, such as carnosine.

Carnosine (β-alanyl-l-histidine) is an endogenous dipeptide that is characterized by a high therapeutic potential, being a well-known antioxidant and also having metal chelating, anti-aggregating, anti-inflammatory, and neuroprotective properties [8,9]. Due to its multimodal mechanism of action, including its ability to improve intramuscular buffering capacity (suppressing acidosis), carnosine has already been considered as a “natural endogenous antidote”.

It is well-known that one of the main causes of muscle fatigue is muscle acidosis, induced by intense exercise, which significantly interferes with the normal production of energy and inhibits muscle contraction [10]. The body fights this problem by releasing natural antidotes such as phosphates and carnosine. The latter has shown the ability to regulate calcium sensitivity in the sarcoplasmic reticulum during musculoskeletal contraction [11], as well as to reduce lactic acid accumulation in muscles during exercise, leading to improved athletic performance [12,13]. There are several additional cases and clinical scenarios in which carnosine can be considered as a potential antidote, particularly when considering its antioxidant activity.

Oxidative stress has been defined as a status in which the production of reactive and pro-oxidant species exceeds the antioxidant defense system [14,15,16]. The endogenous antioxidant machinery protects cells from oxidative stress and related inflammatory phenomena by enhancing the expression of cytoprotective enzymes and is regulated by Nrf2. For this reason, the latter has emerged as a promising new therapeutic target in clinical toxicology [17].

In the present review, we first consider the different pharmacological classes that are known to induce cardiotoxic and neurotoxic effects through the impairment of the Nrf2 pathway, before focusing our attention on the therapeutic potential of carnosine as an antidote that is able to rescue and/or enhance Nrf2 and prevent and/or counteract drug-induced cardiotoxicity and neurotoxicity.

## 2. Pharmacological Classes Involved in Cardiotoxicity and Neurotoxicity

Cardiotoxicity represents a major drug-induced adverse effect that includes clinically important agents such as first-generation antineoplastic drugs (anthracyclines, cyclophosphamide, 5-fluorouracil, and taxanes), monoclonal antibodies (trastuzumab, bevacizumab, and nivolumab). tyrosine kinase inhibitors (sunitinib and nilotinib), antiretroviral drugs (zidovudine), antidiabetic drugs (rosiglitazone), and drugs of abuse [18,19,20,21,22]. In particular, anticancer drugs—mainly anthracyclines, 5-fluorouracil, and cyclophosphamide—exert prominent cardiotoxicity. The main drug that is currently available for the pharmacological treatment of doxorubicin (DOX)-related cardiotoxicity is dexrazoxane, the only recently approved drug that is able to suppress oxidative stress in cardiomyocytes. Other drugs that are commonly used to reduce cardiotoxicity include ACE inhibitors, L-carnitine, probucol, CoQ10, *N*-acetylcysteine, vitamin E, and deferoxamine, but an urgent need remains to develop novel antidotes as pharmacological tools to prevent this clinically relevant adverse drug effects [23].

### 2.1. Drug-Induced Cardiotoxicity

Different studies have demonstrated that drug-induced cardiotoxicity represents one of the major toxic effects that are associated with recent drug discovery processes and drug development, and it is not limited to old drug classes. In particular, chronically administered drugs, including psychotropic drugs and antineoplastic agents, have been shown to cause cardiotoxicity following long-term accumulation [24,25]. However, the advancements in cancer pharmacotherapy combined with improved overall survival have significantly increased the awareness on the adverse cardiac effects of cancer treatment itself [24].

Cardiotoxic effects have been extensively reported in some classes of antidepressants (e.g., tricyclics) and first-generation antipsychotic (FGA) drugs that can lead to severe cardiovascular complications, such as cardiac arrhythmias and death, even in patients with no previous cardiac disease history [26,27,28].

Oxidative stress, free radical generation, and hypoxia play a key role in the pathophysiology of cardiotoxicity [29]. Drugs that induce cardiotoxicity can be classified according to their clinically manifested effects: (1) drugs causing cardiac injury by affecting the performance of cardiac muscles; (2) drugs altering the ion channels and pump (voltage-gated sodium and potassium ion channel and Na-K ATPase pump) [30]. According to this classification, these drugs can be used as cardiotoxicity inducing agents in preclinical models to identify the molecular pathways underlying drug-induced cardiotoxicity [23].

Despite this, concern regarding cardiac safety is growing, representing the main cause for the withdrawal of drugs from clinical trials or, in the worst scenario, from the market. Therefore, if on one hand, the continuous pharmacovigilance system assures the safety of new marketed drugs, on the other hand, the molecular mechanisms underlying cardiotoxicity are not fully understood [31,32,33].

#### 2.1.1. Cardiotoxicity Induced by Central Nervous System (CNS) Agents

*Antidepressants.* The available evidence suggests that tricyclic antidepressants (including amitriptyline, amoxapine, desipramine, doxepin, imipramine, nortriptyline, protriptyline, and trimipramine) represent the most common cause of cardiotoxicity that is associated with the use of antidepressants [34], due to the inhibition of cardiac calcium, sodium, and potassium channels, causing fatal arrhythmias and hypotension in case of overdose [35]. The second-generation antidepressants, such as selective serotonin reuptake inhibitor (SSRIs) and serotonin-noradrenaline reuptake inhibitors, report low-risk cardiac adverse effects, but they can interact with calcium, sodium, and potassium channels [36].

*Local anesthetics.* The main function of local anesthetics is to block sodium channels that regulate the action potential activation on which depend myocardial and nervous tissues [37,38]. In particular, bupivacaine and lidocaine represent the last generation of local anesthetics that are specifically developed to overcome cardiotoxicity. However, clinical evidence showed that bupivacaine is able to exert remarkable cardiotoxic effects compared to other local anesthetics causing common hemodynamic and electrophysiological disorders, as well as hypoxia, arrhythmia, and cardiac arrest. Clinical studies have also demonstrated that lidocaine and ropivacaine can cause dose-dependent adverse cardiac reactions [39,40].

*Antipsychotics.* The cardiotoxic effects of antipsychotics are not limited to the first generation (e.g., thioridazine and haloperidol), because the administration of clozapine, approved for the treatment of resistant schizophrenia, has been associated, in some cases, with adverse cardiac effects such as myocarditis and cardiomyopathy. Ziprasidone and haloperidol can cause serious side effects such as torsade de pointes arrhythmias or even sudden cardiac death [41].

*Drugs approved for neurodegenerative diseases.* In recent years different drugs have been developed for the treatment of Alzheimer’s disease (AD) and Parkinson’s disease (PD), but some of these drugs have shown cardiotoxic effects. For instance, the use of pergolide, a dopamine D2 receptor agonist that is approved for the treatment of PD, has been limited due to the increased risk of fibrotic valvular heart disease [42] related to the ability of this drug to activate the 5-hydroxytryptamine receptor 2B (HTR2B) serotonin receptors and the transforming growth factor beta (TGF-β) pathway [43,44].

*Other psychotropic drugs.* Methysergide and ergotamine, drugs that are highly effective in migraines—primarily by activating 5-hydroxytryptamine 1B (5-HT(1B)), 5-HT(1D), and 5-HT(1F) receptors—have been shown to share toxic effects with pergolide, causing cardiac fibrosis [45] that is related to the activation of the TGF-β pathway through the 5-HT2B receptor. Additionally, some appetite inhibitors, such as dexfenfluramine, sibutramine, and fenfluramine were lifted from the market due to cardiac fibrosis [46].

#### 2.1.2. Cardiotoxicity Induced by Anti-Neoplastic Agents

*Anthracyclines.* Anthracyclines represent the first line anticancer drugs, but their use is reduced due to adverse cardiac effects including systolic and diastolic dysfunction [22,47]. In addition, an important interindividual variability in susceptibility to chronic anthracycline-induced cardiotoxicity has been demonstrated, suggesting that genetic variants have an impact on the onset of drug-induced cardiotoxicity [20,48]. The cardiotoxic effects of these drugs can be the consequence of irreversible microstructural lesions of cardiomyocytes leading to necrosis and apoptosis (type 1 drug-induced cardiotoxicity), mediated by oxidative stress directly or indirectly [49], and reversible cardiotoxicity (type 2) [50,51]. The available evidence emphasizes that free radicals play a key role in the topic of anthracyclines-induced cardiotoxicity [52,53] with nitrite free radicals as the major culprit of oxidative stress, causing both myocardial death and dysfunction in the contraction of the myocardial muscles [47,54,55].

*5-fluorouracil.* Cardiotoxicity that is associated with the use of 5-fluorouracil occurs mainly in the first hours of the initial treatment cycle and may include chest pain, ECG changes, arrhythmia, pulmonary edema, myocardial infarction, and cardiac arrest. Therefore, the administration of this drug in patients with pre-existing cardiac disease is led by careful clinical monitoring and should be stopped immediately in patients who develop cardiac complications [37].

*Methotrexate (MTX).* It is also used at low doses for the treatment of different autoimmune diseases such as rheumatoid arthritis [56]. MTX-induced cardiotoxicity has been connected to oxidative stress and inflammation [57]. Clinical studies have shown that high MTX doses can cause cardiac symptoms and arrhythmias in previously healthy patients; therefore, it requires further clinical investigation [58].

*Cyclophosphamide.* This is an alkylating agent, and its active metabolite (aldophosphamide) is responsible for antitumor activity. Further, it decomposes into phosphoramide mustard and acrolein. Acrolein is a toxic metabolite which acts on myocardium and endothelial cells. Generally, cyclophosphamide-induced cardiotoxicity is associated with the effects of acrolein on endothelial cells that cause severe myopericarditis and myocardial necrosis [59,60], in particular, with a high probability of death within two weeks in patients suffering from congestive heart failure [61].

*Antiretroviral drugs.* Mitochondria play an important role in myocardial tissue homeostasis, and the impairment of their function leads to the death of both cardiomyocytes and endothelial cells and consequent cardiovascular dysfunction [18]. Antiretroviral nucleoside reverse transcriptase inhibitors (e.g., zidovudine) have been shown to cause cardiac mitochondrial dysfunction through the inhibition of DNA polymerase gamma and the induction of mitochondrial DNA mutations causing cardiomyopathy [62].

*Tyrosine kinase inhibitors.* Tyrosine kinase inhibitors have made great progress in the treatment of cancer, but the occurrence of acquired resistance and the development of cardiac effects have been observed [63]. Sorafenib and vandetanib, vascular-endothelial growth factor (VEGF) signaling inhibitors, are known to share adverse cardiac effects with lapatinib, an epidermal growth factor signaling inhibitor, and to cause cardiotoxicity. The primary mechanism of cardiotoxicity induction is believed to involve the inhibition of major signaling pathways that are responsible for cardiomyocyte survival and maintenance [64,65]. Imatinib-induced cardiotoxicity is associated with the release of B-cell lymphoma 2 (Bcl-2-associated X protein), which causes mitochondrial depolarization and damage [64]. Interestingly, imatinib and sunitinib are both able to activate calcium/calmodulin-dependent protein kinase II expression and activity in vitro, but without affecting myocardial contractility [66], and to induce reactive oxygen species (ROS) levels, leading to reduced cell viability [67].

*Biological monoclonal antibodies.* Cardiotoxicity that is induced by monoclonal antibodies is mainly associated with their specific targets [65]. For example, the drug trastuzumab, a potent humanized anti-human epidermal growth factor receptor 2 monoclonal antibody has been shown to downregulate neuregulin-1, a signaling molecule that is essential in cardiac homeostasis and development [68], causing hypertension and even myocardial infarction [69]. Along this line, the use of bevacizumab is associated with the adverse effects on the coagulation system, such as thromboembolism, probably because of its vascular endothelial growth factor receptor (VEGFR) inhibition effects [69].

*Drugs of abuse.* The illegal drug cocaine, an alkaloid that is obtained from the “Erythroxylon coca” plant, acting as a potent inhibitor of dopamine and noradrenaline transporter, is responsible for the release of norepinephrine and epinephrine from the adrenal medulla, causing severe vasoconstriction [70]. Its most common cardiac adverse effects are myocardial ischemia or myocardial infarction [71], tachycardia, and increased systolic-diastolic blood pressure [72]. Chronic cocaine administration causes coronary artery vasoconstriction and thrombosis. Together, these promote myocardial ischemia, whereas the acute use of cocaine causes an increase in the intracellular concentration of calcium and then stimulates arrhythmia [73].

Alcohol abuse negatively affects both the CNS and the heart system [74,75]. In particular, alcohol use exerts direct cardiotoxic effects on myocardial contractility, systolic–diastolic deregulation, and abnormal rhythm [76].

*Other antineoplastic agents*. Bortezomib, a proteasome inhibitor that is approved for myeloma treatment, can cause different side effects including neutropenia, thrombocytopenia, and reversible cardiotoxicity (e.g., arrhythmias and even heart failure) [77,78]. Recently, clinical trials have raised concerns regarding the cardiac safety of immune checkpoint inhibitors and new and effective anticancer therapies [79,80]. In particular, the cardiotoxic mechanism of checkpoint inhibitors has been associated with immune-related disorders, as in the case of vitiligo [81].

#### 2.1.3. Cardiotoxicity Induced by Anti-Inflammatory Agents

Nonsteroidal anti-inflammatory drugs (NSAIDs) represent an important class of agents with anti-inflammatory properties that target one or both prostaglandin synthases (cyclooxygenase-1 (COX-1) and COX-2) [2]. NSAID-induced cardiotoxic side effects such as gastrointestinal toxicity are associated with the inhibition of COX-1 and COX-2 [82]. The primary mechanism of cardiotoxicity induction is believed to involve the blocking of prostacyclin synthase without affecting thromboxane A2 synthesis, causing an increased risk of thrombosis [83].

#### 2.1.4. Cardiotoxicity Induced by Anti-Infective Agents

Recent studies have raised concerns over the cardiac safety of different anti-infective drugs.

*Antibiotics.* Antibiotics, among the most used drugs worldwide, exert the principal function of disrupting the bioactive processes of pathogens [2]. Interestingly, macrolide antibiotics can inhibit bacterial protein synthesis and are widely used for respiratory infections via the inhibition of human ether-à-go-go related gene (hERG) [84]. It is worth mentioning that some macrolides, e.g., erythromycin, azithromycin, and clarithromycin, are considered to be arrhythmogenic [85]. Fluoroquinolones including both grepafloxacin and sparfloxacin represent another major group of antibiotics that are able to affect hERG causing torsade de pointes, leading to their withdrawal from the market [86].

*Antivirals.* Antiviral therapy causes mitochondrial toxicity in liver, skeletal muscle, and heart tissues [87]. A major antiretroviral treatment for HIV is represented by azidothymidine, which can induce mitochondrial dysfunction through mitochondrial fragmentation and an impaired fusion-fission cycle [88]. Furthermore, azidothymidine has been shown to inhibit mitochondrial DNA polymerase, alongside its main target, reverse transcriptase [87]. Recent clinical studies have reported that sofosbuvir, an inhibitor of RNA polymerase nonstructural protein 5B, used to treat hepatitis C, is able to induce cardiotoxicity [89].

*Other anti-infective agents.* Pentamidine, commonly used for the treatment of leishmaniasis, trypanosomiasis, and pneumonia, causes cardiac adverse effects such as QT prolongation and ventricular arrhythmias in intravenous treatment [90]. In vitro experiments documented that pentamidine is able to inhibit hERG trafficking, causing action potential prolongation in both animal and human cells [91,92], despite being a poor direct blocker of IKr at therapeutic concentrations [93].

### 2.2. Drug-Induced Neurotoxicity

Neurotoxicity is primarily linked to complex interactions of xenobiotics at the molecular, cellular, and tissue level of the central and/or peripheral nervous system, responsible for major adverse effects such as changes in neuronal and/or glial cell structure and/or function. Therefore, several strategies have been proposed to develop new antidotes against drug-induced neurotoxicity based on the combination of relevant in vitro models [94,95]. Public databases and searches of the literature propose four different categories of CNS-directed drugs that induce neurotoxicity: (1) neuroactive and neurotoxic: CNS drugs with significant neurotoxic effects (amiodarone, buflomedil, chlorpromazine); (2) neuroactive and non-neurotoxic: CNS drugs with weak neurotoxic effects (carbamazepine, diazepam, propofol); (3) non-neuroactive but neurotoxic: non-CNS drugs with severe neurotoxic effects (cisplatin, ciprofloxacin, cyclosporine A (CsA)); (4) non-neuroactive and non-neurotoxic: non-CNS drugs with weak or absent neurotoxic effects (loperamide, nadolol, ondansetron). Among the selected drugs, we cannot forget that diazepam, which is not neurotoxic after acute exposure, can exert clinically relevant neurotoxic effects after long-term exposure [96]. It is worth noting that neurotoxicity is linked to its primary target, the blood–brain barrier, which is essential both to maintain the homeostasis of the brain [97,98] and to limit the entry of most molecules into the CNS, preventing toxicological effects in the brain [99]. Therefore, in vitro tests to evaluate compound-induced neurotoxicity must predict whether a drug will reach the CNS in amounts that are sufficient to cause toxicity [100,101].

#### 2.2.1. Neurotoxicity Induced by Anti-Neoplastic Agents

Antineoplastic agents present the lowest therapeutic index compared to other drugs, causing frequent and predictable multisystem toxicity [102]. Vincristine represents the most important antineoplastic drug exhibiting dose limiting neurotoxicity effects. Vincristine can cause neuropathic pain with paresthesia of the hands and feet and the loss of deep tendon reflexes and weakness. Specifically, subjects with diabetes, alcohol abuse, and those with inflammation and toxic neuropathy have an increased vulnerability with a high risk of neurotoxicity [103]. Drugs that induce neurotoxicity can be classified according to the induced clinical phenotype: (1) acute myelopathy: intrathecal cytarabine, intrathecal methotrexate, and intrathecal thiotepa; (2) autonomic neuropathy: cisplatin, paclitaxel, procarbazine, vindesine, vinblastine, and vincristine; (3) encephalopathy: carmustine, procarbazine, cisplatin, cytarabine, 5-flurouracil, and ifosfamide; (4) cerebellar syndrome: cytarabine, procarbazine, and 5-flurouracil; (5) cranial nerve toxicity: vindesine, carmustine, vinblastine, cisplatin, vincristine, and ifosfamide; (6) peripheral neuropathy: vincristine, carboplatin, vinblastine, procarbazine, vindesine, and paclitaxel [104]. Regarding MTX, it is a widely used chemotherapic agent, especially for the treatment of pediatric hematological cancers. MTX has been shown to cause neurotoxicity by inducing white matter damage [105]. MTX has also been associated with clinical neurotoxicity and asymptomatic leukoencephalopathy [106].

#### 2.2.2. Neurotoxicity Induced by Antipsychotic Drugs

Different studies have demonstrated that antipsychotic drugs, specifically first-generation agents, are associated with neurotoxic effects and a decline in gray matter volume [107]. For this reason, the possible neurotoxic mechanisms of FGA, particularly haloperidol, have been reviewed [108]. Haloperidol is a butyrophenone, better known as a neuroleptic agent, that is used in the treatment of schizophrenia and delirium and is known to induce severe adverse effects such as extrapyramidal symptoms (EPS) [108]. Several published studies have reported that haloperidol exerts neurotoxic effects through different molecular mechanisms, leading to neuronal death. A similar result was reported by FGA for fluphenazine and perphenazine; in contrast, second-generation agents (SGAs), such as aripiprazole, paliperidone, and risperidone, showed a protective action against brain loss in schizophrenia [107]. In particular, the increased affinity to 5HT-2A receptors compared to dopamine D2 receptors contribute to the neuroprotective effect among SGAs [109]. The oxidative stress that is induced by haloperidol plays a key role in the development of extrapyramidal side effects. Haloperidol also exerts neurotoxic effects by decreasing antioxidant enzyme levels, then worsening pro-oxidant events [110]. Different clinical trials have demonstrated a synergistic neurotoxic effect of haloperidol in combination with lithium, a mood stabilizer, with the clinical onset of EPS, seizures, ataxias, hyperreflexias, and abnormal EEG’s whose pathophysiology remains unclear [111].

In addition, the combined use of lithium and risperidone, an atypical antipsychotic drug, has also been demonstrated in the treatment of psychotic disorders, showing neurotoxic and nephrotoxic events in patients with bipolar or schizoaffective disorder [112]. Severe adverse drug reactions have been reported in multiple organ systems with their combined use. These complications resolved soon after the discontinuation of the drugs and adequate hydration [113].

#### 2.2.3. Neurotoxicity Induced by Immunosuppressive Drugs

Commonly used immunosuppressive drugs for the prevention of transplant rejection are tacrolimus, CsA, and mycophenolate mofetil [114]. CsA does not suppress bone marrow and its potential side effects include nephrotoxicity, hypertension, gingival hyperplasia, hypertrichosis, infection, hyperkaliemia, hypomagnesaemia, hepatotoxicity, increased incidence of specific tumors, and neurotoxicity [115,116,117]. Generally, CsA-induced neurotoxicity is associated with intravenous administration and high dose, the administration of CYP450 inhibitors, and liver transplantation, but the underlying mechanism is unclear to date [118]. Regarding mycophenolate mofetil, it can cause neurological side effects such as headache, insomnia, dizziness, depression, confusion, hypertonia, and paresthesia [119]. Computed tomography scanning and magnetic resonance imaging represent the main tools for evaluating radiological abnormalities in patients with CsA-induced neurotoxicity [120], such as signal changes in the cerebral cortex and juxta-cortical white matter of the occipital lobes, temporal, parietal, and frontal lobes [114].

Tacrolimus is the most used immunosuppressive drug for the prevention of transplant rejection and its use has been associated with neurotoxic effects such as tremor, headache, seizures, delirium [121,122], and encephalopathy, especially in pediatric populations [123]. The molecular pathways underlying neurotoxicity are presently unknown, although it is well-known that tacrolimus can inhibit antioxidant enzymes and impair the Nrf2 pathway [124].

## 3. The Nrf2 Pathway

Oxidative stress and the related pro-inflammatory phenomena play a pivotal role in both the initiation and progression of numerous chronic diseases, including diabetes, cancer, and cardiovascular and neurodegenerative diseases [125,126,127,128,129]. A key cellular antioxidant response against oxidative stress due to the over-production of ROS and reactive nitrogen species (RNS), as well as to certain xenobiotics, is given by the intracellular Kelch-Like ECH Associated Protein 1 (Keap1)-Nrf2 response pathway [130]. Nrf2 belongs to the Cap’n’Collar (CNC) transcription factor family and is composed of 605 amino acids (aa) that are divided into seven domains, characterized by different length and function, known as Neh1, Neh2, Neh3, Neh4, Neh5, Neh6, and Neh7 [131]. Regarding the specific function of each domain, Neh2 (the N-terminal one, aa 16–89) modulates the ubiquitination Keap1-dependent of Nrf2, while the cytoplasmic localization of Nrf2 is attributable to the Neh5 (aa 182–209) domain [132]. The longest domain (aa 435–569), Neh1, contains a leucine zipper sequence and a nuclear localization signal (NLS) which are responsible for the regulation of the binding of Nrf2 to DNA [133] and for the nuclear translocation of Nrf2 [134], respectively. Three different domains, Neh3 (the C-terminal one, aa 569–605), Neh4 (aa 111–134), and Neh5 are able to mediate the interaction of Nrf2 with other co-activators (transactivation process) [135,136]. Neh6 (aa 337–394) contains numerous serine residues that play an important role in the negative regulation of Nrf2 (ubiquitination) by binding a β-transducin repeat-containing protein (β-TrCP) [137]. The binding of Nrf2 to the retinoic X receptor α (RXRα), mediated by the Neh7 (aa 209–316) domain, leads to the inhibition of Nrf2/antioxidant response elements (AREs) [138].

Compared to Nrf2, Keap1 is a larger protein (624 aa vs. 605 aa), though it contains a lower number of domains (5 vs. 7): (1) a N-terminal region (NTR) (aa 1–61); (2) a domain that is rich in cysteine residues which are reactive towards ROS, RNS, and electrophiles called Tramtrack and Bric-à-Brac (BTB) (aa 61–180); (3) an intervening region (IVR) (aa 180–356), representing the “central” domain, that contains a nuclear export signal (NES) responsible for the cytoplasmic localization of Keap1 [139]; (4) a C-terminal domain (CTR) (aa 599–624); (5) the largest domain (aa 315–599), composed of six Kelch repeats, mediating the binding of Keap1 to Nrf2 and p62 [140,141], an ubiquitin-binding protein acting as an autophagy cargo receptor [142]. The S-sulfenylation, S-nitrosylation, or S-sulfhydration chemical modifications of specific “sensor” cysteine residues (151 of the BTB domain; 273 and 288 of the IVR domain) that are caused by ROS, RNS, and/or other reactive species have been shown to induce changes in Keap1 conformation, thus leading to its dissociation from Nrf2 [141,143,144].

**Figure 1:**
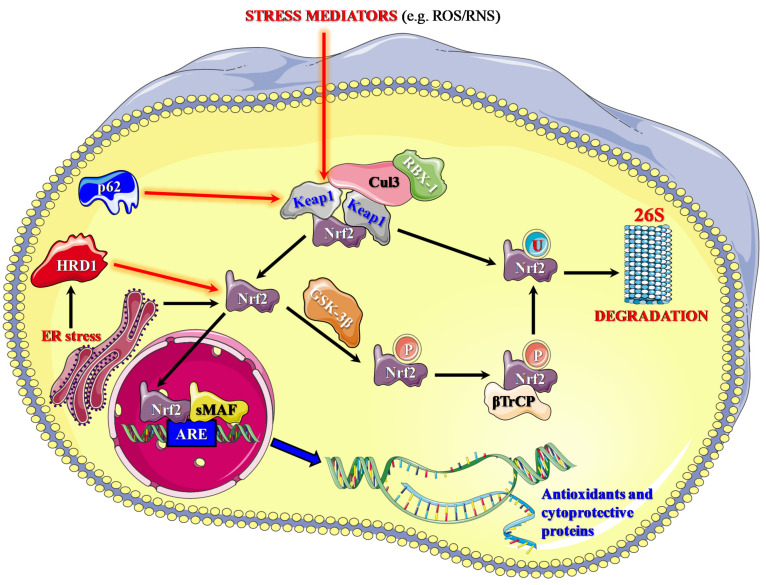
Schematic representation of Nrf2 pathway and its regulatory mechanisms. 26S = proteasome 26S; P = phosphorylation; U = ubiquitination. Black arrows indicate “activation”, while red arrows indicate “inhibition”. Part of the figure has been generated by using Servier Medical Art available at smart.servier.com (accessed on 15 May 2022).

Under physiological conditions, Keap1 strictly controls Nrf2 activity and stability [145,146] (Figure 1).

Nrf2 is then bound to Keap1 and targeted for ubiquitination and proteasomal degradation, with a t_1/2_ of less than 20 min preventing the unnecessary expression of Nrf2 target genes.

Keap1 homodimerization is attributable to the presence of the BTB domain, mediating the binding to the cullin-based (Cul3) E3 ligase [147]. Keap1, under the form of a homodimer, is then able to interact with Cul3, leading to the formation of Keap1-Cul3-ring box protein-1 (RBX-1) E3 ligase complex, which induces the polyubiquitination and consequent degradation of Nrf2 by proteasome (26S) [148]. During the switch from physiological to oxidative stress conditions, the modification of cysteine 151 plays a key role in the conformational alteration and inactivation of Keap1 [149]. At this point, Nrf2 is free to translocate into the nucleus where it forms a heterodimer with small MAF (sMAF) protein, acquiring the ability to bind to the AREs (Figure 1) [145]. Emerging evidence has also revealed several mechanisms of Nrf2 negative regulation that are Keap1-independent [150]. In one of the proposed mechanisms, Nrf2 is negatively regulated by the glycogen synthase kinase 3β (GSK-3β), an enzyme which plays a key role in apoptotic cell death. GSK-3β phosphorylates two different serine residues (342 and 347), a key event allowing the binding of Nrf2 to the E3 ubiquitin ligase complex β-TrCP, leading to its ubiquitination and degradation [137,151]. An alternative Keap1-indipendent mechanism is mediated by the activity of E3 ubiquitin ligase HRD1 (also known as synoviolin) [152]. In response to endoplasmic reticulum (ER) stress, there is a transcriptional up-regulation of HRD1 which is able to directly interact with Nrf2, promoting its ubiquitination and proteasomal degradation [152]. Under physiological conditions, the substrate p62 is constantly degraded during the autophagy process; however, when oxidative stress occurs, p62 accumulates and directly interacts with Keap1, competitively inhibits its binding to Nrf2, then protects Nrf2 from Keap1-mediated degradation [153].

Nrf2 is able to regulate the transcriptional induction of numerous genes that are related to glutathione (GSH) (glutamate–cysteine ligase complex, glutathione reductase 1, and cystine-glutamate antiporter system x_c_^−^) and thioredoxin (thioredoxin reductase 1 (Trx-1) and sulfiredoxin) production, utilization, and regeneration; NADPH regeneration (glucose-6-phosphate dehydrogenase, 6-phosphogluconate dehydrogenase, isocitrate dehydrogenase 1, and malic enzyme 1); heme (heme oxygenase 1 (HO-1)) and iron (ferritin light polypeptides and heavy polypeptides) metabolism; ROS, RNS, and xenobiotic detoxification (glutathione peroxidase 2, glutathione S-transferases (GSTs); and NADPH quinone dehydrogenase 1 (NQO1)), providing the main intracellular cytoprotective defense system [154,155,156].

Nrf2 and the nuclear factor kappa-light-chain-enhancer of activated B cells (NF-κB) pathways cooperate with each other to maintain the physiological homeostasis of cellular redox status and to regulate the cellular response to stress and inflammation [157]. Regarding NF-κB, it is not a single protein, but a small family of inducible transcription factors regulating the expression of genes that are implicated in immune response (innate and adaptive), inflammation, and oxidative stress, as well as B-cell development [158,159]. From a functional point of view, Nrf2 is able to exert the negative regulation of the NF-κB pathway by different mechanisms: (1) decreasing the intracellular levels of ROS and RNS and then the oxidative stress-mediated activation of NF-κB [160]; (2) preventing the proteasomal degradation of the nuclear factor of kappa light polypeptide gene enhancer in B-cells inhibitor, alpha (IκB-α) and inhibiting the nuclear translocation of NF-κB [161]; (3) up-regulating the expression levels of HO-1 levels, thus blocking the degradation of IκB-α [162]; (4) competing with the transcription co-activator cAMP response element (CREB) binding protein (CBP) [42]. It is worth mentioning that some Nrf2 target genes such as NQO1 are also believed to possess a NF-κB binding site, a reason why it can be supposed that in specific circumstances these two pathways may cooperate [163,164].

## 4. Nrf2 Pathway Modulation and Its Connection with Cardiotoxicity and Neurotoxicity

Cardiovascular diseases (CVD) represent the leading cause of death worldwide with several conditions being affected by oxidative stress [165]. The increased production of ROS and RNS has been linked among others to arterial hypertension and arrhythmia. The use of different drugs has been associated with oxidative stress, inflammation, and the related cardiotoxicity, as has already been shown by numerous preclinical in vitro and in vivo studies (including gene knockout (KO) approaches [166]), showing the key of the Nrf2/ARE system and/or the rescue of this pathway as a novel strategy to develop antidotes.

Along this line, preclinical studies with MTX have demonstrated the link between lipid peroxidation, NF-κB activity, inflammatory markers, decreased antioxidant enzymes activity, and anti-inflammatory markers (e.g., IL-10) with the impairment of the Nrf2 pathway [167]. In the same study, the authors demonstrated that the protective effect of vincamine against MTX-induced nephrotoxicity was mediated by the induction of both Nrf2 and HO-1.

Similar data have been obtained with clozapine analyzing the proteome of human oligodendrocytes (MO3.13), where the toxicity pathways that were generated by the analysis were associated mainly with Nrf2, oxidative stress, and cell cycle G2/M DNA damage in response to clozapine treatment [168].

Regarding 5-fluorouracil, this chemotherapeutic agent has been shown to give toxicity, mainly increasing the expression of Keap1 and decreasing Nrf2 transcriptional activity, as well as to the inhibition of HO-1 and the aggravation of DNA damage [169].

It is well-known that the anthracycline DOX, extensively used for the management of breast cancer, lymphoma, and hematologic malignancy [170], has been connected to serious cardiotoxic side effects, a reason why this molecule is often used in preclinical studies to induce cardiotoxicity, as well as to study the therapeutic potential of molecules of interest. Of note, numerous studies have clearly shown both in vitro and in vivo that the observed drug-induced cardiotoxicity is often paralleled by the impairment of the Nrf2 pathway. Very recently, Li et al. have shown that fisetin, a natural flavonoid that is abundantly present in fruits and vegetables, exerts its therapeutic effects against DOX-induced cardiomyopathy by inhibiting ferroptosis via Sirtuin 1 (Sirt1)/Nrf2 signaling pathway activation [171]. Fisetin was able to improve the myocardial function by alleviating cardiac dysfunction, improving myocardial fibrosis, and decreasing cardiac hypertrophy in rats that were challenged with DOX, while increasing the expressions of the Sirt1/Nrf2 pathway genes, HO-1, and ferritin heavy chain 1 in rats, as well as H9c2 myoblasts. In a different research study, Zhang et al. demonstrated that the activation of Nrf2 (and down-regulation of Keap1 negative modulator) by using the novel activator miR-152 inhibited the cardiotoxicity that was induced in mice by DOX drug treatment attenuating oxidative stress, inflammation, and apoptosis [172]. Tanshinone IIA is a lipophilic active constituent that is obtained from the roots and rhizomes of *Salvia miltiorrhiza* Bunge (Danshen), a Chinese medicinal herb [173] that has been reported to protect against DOX-induced cardiotoxicity. In the animal model of DOX-induced acute cardiotoxicity, the pre-treatment with tanshinone IIA was able to reduce the activity of myocardial enzymes, also increasing the activity of superoxide dismutase (SOD) and catalase antioxidant enzymes, as well as GSH levels [174]. When investigating the molecular mechanisms in vitro and n vivo, tanshinone IIA pre-treatment was shown to induce the nuclear translocation and accumulation of Nrf2 and its downstream genes HO-1, NQO1, and glutamate-cysteine ligase catalytic subunit (GCLC) in the cardiac tissues of mice and H9c2 cells. The key role that was played by Nrf2 in the observed cardioprotection against DOX was also demonstrated by using a small interfering RNA able to knock down Nrf2. In addition to tanshinone IIA, numerous natural compounds such as asiatic acid (↑ Nrf2 nuclear translocation and ARE activity; ↓ Keap1 expression); α-linolenic acid (↑ Nrf2 (nuclear) and Keap1 (cytosolic) levels); apigenin (improved cellular redox defense in a concentration-dependent manner via regulation of the Nrf2/HO-1 pathway); baicalein (blocked Nrf2 binding to Keap1; ↑ p62 expression; sustained phosphorylation of extracellular-signal-regulated kinase 1/2 (ERK1/2) and protein kinase C (PKC)); β-LAPachone (↓ NF-kB; ↑ Nrf2, HO-1, and GST); curdione (↑ Nrf2 and HO-1); dioscin (↑ Nrf2/ARE; ↑ NQO1, HO-1, and GST expression levels); ferulic acid (↑ Nrf2/HO-1 pathway); *G. lucidum* polysaccharides (↑ Nrf2 and HO-1); genistein (regulation of PKC/Nrf2/ARE pathway); ginsenoside Rg3 (↑ Akt/Nrf2/ARE pathway); indole-3-carbinol (Nrf2-mediated activation of the ARE pathway; ↑ HO-1 and NQO1); naringenin-7-O-glucoside (↓ Nrf2-Keap1 interaction; ↑ ERK, Nrf2, and NQO1); neferine (↑ Nrf2 translocation and SOD and HO-1 expression; induction of insulin-like growth factor 1/phosphatidylinositol 3-kinase (PI3K)/Akt/Nrf2 pathway); p-coumaric acid (activated Nrf2; induced antioxidant enzymes and HO-1 expression); pristimerin (↑ Nrf2/ARE pathway); punicalagin (activated PI3K/Akt/Nrf2 pathway); quercetin (↑ Nrf2); and sulforaphane (altered Keap1 conformation; blocked Nrf2 ubiquitination and degradation; increased Nrf2 translocation to the nucleus) have shown the ability to counteract the cardiotoxicity that is induced by DOX by rescuing and/or activating Nrf2 and related pathways [175]. Still in the context of DOX-induced cardiotoxicity, Sharma et al. recently demonstrated that the increased expression of Nrf2 mRNA and then the enhancement of antioxidant defense that was induced by quercetin administration was able to prevent biochemical and histological abnormalities in rats [176].

Cyclophosphamide is known to cause redox imbalance and its use has also been associated with marked cardiotoxicity, with both factors limiting its clinical applications. As observed in the case of DOX, this drug has been shown to exert its cardiotoxicity through the down-regulation of the Nrf2 pathway [177]. Specifically, cyclophosphamide (200 mg/kg) administration induced an up-regulation of the expression of Keap1 mRNA and a down-regulation of Nrf2 both at gene and protein levels in the heart of rats that was paralleled by oxidative stress and cardiac histological alterations. Edaravone and acetovanillone were able to up-regulate Nrf2 along with the PI3K/Akt/mTOR pathway, preventing cyclophosphamide-induced cardiotoxicity in rats, underlining, once again, the key role that is played by Nrf2 in counteracting cardiotoxicity, as well as in maintaining heart homeostasis.

Iron oxide nanoparticles have been used in the biomedical field for numerous purposes, including the enhancement of magnetic resonance images, gene therapy, drug delivery, as well as to elevate body temperature [178]. Unfortunately, this type of nanoparticle has also been reported to cause extensive necrosis through the production of ROS, RNS, and cardiotoxicity [179]. Elgharabawy and co-workers showed the role that was played by the phytochemical activation of Nrf2 in protecting cardiomyocytes from iron oxide nanorod overload-induced cardiotoxicity [180].

Cisplatin represents one of the most commonly used first-line drugs for the treatment of epithelial tumors [181]. Despite this, its clinical use is often limited due to the related side effects including cardiotoxicity and neuropathy [182]. In a very recent publication that was carried out by Jia et al., the cardioprotection exerted by hesperidin, an abundant and economical dietary bioflavonoid, against cisplatin-induced cardiotoxicity in mice was attributed to the regulation of the p62 (rescue)/Keap1 (down-regulation)/Nrf2 (rescue) pathway, leading to the inhibition of oxidative stress, inflammation, and apoptosis [183].

As observed in the case of cardiotoxicity, drug-induced neurotoxicity is often connected to the negative regulation of the Nrf2 pathway. However, it is worth pointing out that compared to cardiotoxicity, fewer studies have been carried out to study the link between drug-induced neurotoxicity and the negative regulation of Nrf2.

The antineoplastic chemotherapy drug vincristine has been found to promote the up-regulation of NF-κB levels and the suppression of the Nrf2 pathway in sciatic nerves causing oxidative stress and neuroinflammation [184].

Paclitaxel has been shown to cause peripheral neuropathy. As shown by Miao et al., this drug can lead to Nrf2-ARE and SOD impairment in the dorsal root ganglion, also increasing several oxidative stress mediators, such as 8-isoprostaglandin F2alpha and 8-hydroxy-2′-deoxyguanosine [185].

Epithelial-mesenchymal transition (EMT) has been implicated in immunosuppressive CsA-induced renal fibrosis. The potential role of the Nrf2/HO-1 system on the regulation of CsA-induced EMT-renal fibrosis has been investigated [186]. The pre-treatment of rat tubular epithelial NRK-52E cells with sulforaphane, an activator of the Nrf2 pathway, prevented EMT gene changes. Overall, their results suggested that the Nrf2-HO-1 system played a protective role against CsA-induced renal fibrosis by modulating EMT gene changes.

Recently, it has been observed that astragaloside IV (AS-IV), a saponin extract of Astragalus, is able to relieve chronic tacrolimus-induced nephrotoxicity through the modulation of the p62-Keap1-Nrf2 pathway. In particular, AS-IV increased p62 phosphorylation leading to Nrf2 nuclear translocation, then preventing ROS accumulation and renal fibrosis, a clinically relevant adverse effect that is induced by tacrolimus [124].

Ifosfamide represents a synthetic analogue of cyclophosphamide that is used for the treatment of various solid cancers. In a study that was carried out by Han et al., the intraperitoneal (i.p.) administration of ifosfamide in rats was linked to severe renal toxicity, as well as to the down regulation of Nrf-2 mediated stress oxidative response pathways [187].

PD represents the second most common neurodegenerative disease among people over 60 years of age worldwide and is characterized by oxidative stress that significantly contributes to the selective neurodegeneration of dopaminergic neurons in the *substantia nigra* [188]. 6-hydroxydopamine (6-OHDA) is a neurotoxic compound, able to induce oxidative stress, that has been extensively used to mimic an early stage of PD in different experimental models in vitro and in vivo. By employing a PD model consisting of SH-SY5Y cells challenged with 6-OHDA drug, Ma et al. demonstrated that the observed 6-OHDA-induced oxidative stress and neurotoxicity were connected to the significant deregulation of the Nrf2 pathway; specifically, 6-OHDA significantly inhibited the expression of Nrf2, along with its downstream products, GCLC), glutamate-cysteine ligase modifier subunit (GCLM), HO-1, NQO1, and Trx-1, as well as the phosphorylation of AMP-activated protein kinase and Akt in SH-SY5Y cells [189]. Moving to an in vivo 6-OHDA-induced PD model, the Nrf2-mediated neuroprotection that is exerted by icariin has been demonstrated [190]. Icariin was able to rescue Nrf2 signaling activation in a 6-OHDA-induced mouse PD model, while failed to generate dopamine neuroprotection and abolish glial cells-mediated inflammation in Nrf2 KO mice.

A substance whose chronic use is associated with PD and neurotoxicity is represented by 1-methyl-4-phenyl-1,2,3,6-tetrahydropyridine (MPTP). A recent paper describes the nigrostriatal dopaminergic neurons toxicity due to 1-methyl-4-phenylpyridinium (MPP+) and MPTP [191]. In this study, the authors were able to demonstrate that hydralazine, an approved drug by the U.S. Food and Drug Administration (FDA) for antihypertensive treatment, protected against the neurotoxicity that was induced by MPP+ and MPTP, with a pivotal role played by the activation of the Nrf2-ARE signaling pathway. It was then hypothesized that the neuroprotective activity hydralazine-mediated was obtained by acting as a potent Nrf2 activator, with a consequent increase in the expression of Nrf2 downstream ARE target genes. It has been demonstrated that acrylamide group can be used to improve the drug-like properties of potential drug candidates [192]. It has also been demonstrated that acrylamide is a well-characterized neurotoxic agent; indeed, it is known to induce neuropathy and encephalopathy in both human beings and experimental animal models [193]. The role of Nrf2 activation in attenuating acrylamide-induced neuropathy in male C57Bl/6JJcl adult mice has been recently confirmed. In this study, acrylamide dose-dependently and significantly increased the degeneration of monoaminergic axons in the S1HL, S1FL, S1BF, and S2 regions, and the observed neurotoxicity was connected to the down-regulation of Nrf2 and related genes (SOD-1, NQO1, glutathione S transferase mu, and metallothionein 1), as well as to the exacerbation of pro-inflammatory mediators (tumor necrosis factor-α (TNF-α) and inducible nitric oxide synthase). In the same study, the sulforaphane-mediated induction of the Nrf2 pathway led to protection against acrylamide-induced neurotoxicity, strengthening the relevance of this factor and its downstream ARE genes. The above-mentioned results were “indirectly” strengthened by Ekuban et al., demonstrating that the genetic ablation of Nrf2 enhances acrylamide-induced sensorimotor dysfunction and monoaminergic axon degeneration, along with microglial activation in mouse prefrontal cortex [194]. These studies suggest a central role of Nrf2 pathway rescue/activation in counteracting both oxidative stress and inflammation, related to neurotoxicity.

Alcohol is a psychoactive drug that is known to cause CNS and liver injury [195]. Hepatic Nrf2 has been shown to play a key role in the development of the morbidity and liver injury in vivo. By using an alcoholic liver disease mouse model, Sun et al. showed that liver-specific Nrf2 deficiency accelerates ethanol-induced lethality and hepatic injury [196]. From the molecular point of view, Nrf2 induces the expression of ethanol detoxification enzymes, contributing to an increase in sensitivity to the toxicity that is induced by ethanol. Ethanol is also often used to induce neurotoxicity in both in vitro and in vivo systems. In a study that was carried out by Ali et al., the Nrf2 gene-dependent antioxidant mechanisms underlying the possible neuroprotective effects of melatonin supplementation against acute ethanol-induced oxidative stress-mediated neuroinflammation and neurodegeneration in the developing rodent brain were explored [197]. Ethanol decreased the protein expression levels of Nrf2 nuclear fraction, HO-1, and GCLM, and increased Nrf2 cytoplasmic fraction in the cortices and CA1 regions of the hippocampi in the rat pups. Of note, all these alterations were reversed by acute melatonin treatment.

Overall, the above-mentioned studies suggest that drugs can induce cardiotoxicity and/or neurotoxicity through the negative modulation of the Nrf2/ARE pathway, evidencing that Nrf2 can be considered as a novel pharmacological target for the clinical prevention of these adverse drug effects.

According to this new and exciting scenario, we turn in the next section to carnosine, examining its therapeutic potential as an antidote that is able to counteract drug-induced cardiotoxicity and neurotoxicity through the positive regulation of the Nrf2 pathway.

## 5. The Therapeutic Potential of Carnosine as an Antidote in Preventing Drug-Induced Cardiotoxicity and Neurotoxicity through the Modulation of Nrf2

In this section, we briefly describe carnosine’s biological and physiological roles, highlighting its therapeutic potential and multimodal mechanism of action before focusing our attention on its ability to activate the Nrf2 pathway.

The naturally occurring dipeptide carnosine was discovered more than 100 years ago [198,199]. As previously mentioned, carnosine is formed starting from β-alanine (synthesized in the liver) and L-histidine (an essential amino acid) thanks to the enzymatic activity of carnosine synthase 1 (CARNS1) [200,201]. Carnosine can be found at a very high concentration in different tissues such as the cardiac and skeletal muscles (up to 20 mM [202]), containing ~99% of total carnosine [11,203], and the brain [204]. The regulation of the carnosine levels in both human tissues and biological fluids is attributable to the activity of serum-circulating (carnosine dipeptidase 1 (CNDP1) [205]) and cytosolic (carnosine dipeptidase 2 (CNDP2)) [206] carnosinases, two enzymes that are able to metabolize carnosine in its constituting amino acids.

The biological and physiological roles of carnosine and the related therapeutic potential are very broad. The first thing worth noting about carnosine is that, despite its very high levels in muscles and brain, it can exert key biological activities even very far from these tissues, as is well documented by the plethora of research papers that were published during the last two decades. Among them, numerous studies were devoted to the demonstration that carnosine acts as a “natural antidote” in muscle lactic acid detoxification. Specifically, it has been demonstrated that the supplementation of carnosine/anserine is able to prevent intramuscular acidification by reducing lactate accumulation in active muscles [207,208]. In the same context, carnosine has shown the ability to improve the strength of muscle contraction and the mechanical work that is produced (an effect which is better known as “Severin’s phenomenon” [209]) to increase the contraction and relaxation rates of muscles, along with physical performance [210], and to enhance the executive function following endurance exercise [211,212,213,214]. In addition to the above, there are numerous in vitro and in vivo studies reporting the ability of carnosine to: (1) act as an endogenous neurotransmitter [215]; (2) modulate cell energy metabolism by rescuing and/or enhancing the levels of triphosphates and electron carriers [216,217]; (3) regulate the activity of immune cells such as macrophages and microglia [218,219]; (4) increase the degradation rate/scavenging of nitric oxide and related species [220,221,222]; (5) act as an anti-glycation and anti-aging agent [223,224]; (6) chelate transition metals such as copper and zinc [225,226]; (7) up-regulate glutamate transporter 1 and down-regulate the glutamate levels at CNS level [227].

Despite the numerous above-described carnosine activities, the physiological role of carnosine is still not fully understood and should receive more attention. In this regard, a substantial aid could be derived from different recent research studies employing a CARNS1 KO model. For example, Eckhardt et al. have shown that mice KO for CARNS1 have normal skeletal muscle and olfactory function, even though a reduced olfactory sensitivity is observed [228]. It has also been shown that CARNS1^-/-^ rats, characterized by the complete absence of carnosine and its methylated analog, do not present significant changes in terms of exercise capacity, skeletal muscle force production, fatigability, or buffering capacity, strongly suggesting that these are not essential for pH regulation and function in skeletal muscle. On the other hand, KO rats were characterized by a significant impairment of contractile function at cardiac muscle level, underlining the pivotal role exerted by carnosine as a regulator of excitation–contraction coupling in cardiac muscle [229]. Lastly, as demonstrated by Wang-Eckhardt et al., the missing synthesis of carnosine at the endogenous level does not seem to influence protein carbonylation and advanced lipoxidation end products formation in muscle, brain, or kidney [230].

It is significantly worth mentioning that, when performing in vivo studies, a possible drawback could be represented by the animal model that is selected [231]. In fact, due to the presence of CNDP1 and CNDP2, the administration of carnosine in human beings only leads to a small increase in circulating carnosine, limiting its therapeutic potential. Rodents lack the signal peptide in the CNDP1 gene (CTG)_5_, which is the reason why they do not have CNDP1 [11]. This key difference could, at least in part, explain why the levels of carnosine increase in rodents (which are used in more than 100 studies with carnosine), following oral carnosine supplementation [232], something that unfortunately does not take place in humans [233]. The results that are obtained in rodents could then represent an overestimation of the therapeutic potential of this naturally occurring dipeptide.

Moving to clinical trials, the dipeptide carnosine has already been considered for the treatment of different pathological conditions such as mild cognitive impairment [234], depression [235], and type 2 diabetes mellitus (T2DM) [236]. When considering the methodology and study design that are adopted in the numerous clinical trials carried out employing carnosine administration, individually considered or in combination with β-alanine or carnosine’s analogs (e.g., anserine), a significant heterogeneity can be detected in terms of both the formulations and dosages that are selected. Different factors influence the above-mentioned heterogeneity, such as the variability of the subjects that are considered, the intervention types, and the outcomes that are selected, along with the study design and risk of bias. Two very recent systematic reviews with a meta-analysis on the therapeutic potential of carnosine/anserine supplementation against cognitive decline [199] and on the muscle carnosine response to β-alanine supplementation [237], respectively, depict the strong heterogeneity regarding carnosine human studies, as well as the evidence on its clinical efficacy. In the first systematic review [199], it was possible to include only 5 out of 516 studies, while only three provided enough data to be used for the quantitative analysis. In the case of Rezende et al., 7820 records were identified via database searching, but, after a deep analysis, considering the exclusion criteria (e.g., absence of muscle carnosine measurements, use of a supplementation route different from the oral one, not conducted an isolate β-alanine intervention), only 28 studies were considered to be eligible for the meta-analysis, pointing out once again the different characteristics and/or quality of the studies.

As discussed by Calabrese et al., there are numerous dietary antioxidants, including curcumin, acetyl-L-carnitine, and carnosine, that are able to counteract different neurotoxicity through the activation of redox-sensitive intracellular pathways such as Nrf2 [238]. Carnosine has also been shown to modulate, at least in part via Nrf2, oxidative stress and inflammation in the kidney–brain axis, the dysregulation of which contributes to the high prevalence of neuropsychiatric disorders, cognitive impairment, and dementia during the natural history of chronic kidney disease [239]. The modulation of Nrf2 by a dietary antioxidant such as carnosine has also been connected to the prevention of CVD [166,240].

When considering carnosine as a potential antidote that is able to protect through the modulation of the Nrf2 pathway [241], it is important to underline that numerous preclinical studies have shown how this dipeptide seems to be non-toxic [242] and very well tolerated by human beings [243,244], without known drug interactions and toxic side effects. Despite this, there are several studies showing that the administration of carnosine or its precursor β-alanine could lead to unwanted effects. In this context, a study that was carried out by Dolu and co-workers suggested that high-dose carnosine produced anxiety-like effects in rats [245]. In line with these findings, different clinical trials have shown that the administration of β-alanine is linked to paresthesia [246] or an increased perception of pain [247].

It is also worth mentioning that very recently, a bidirectional relationship between carnosine and Nrf2 activation has been proposed [248]. In this research study that was carried out by Ryan et al., by using high-resolution mass spectrometry, respirometry, and metabolomics, it was shown that Nrf2 activation in bone marrow-derived macrophages significantly increased antioxidant metabolites, including carnosine, whereas Nrf2 disruption decreased the intracellular levels of GSH, taurine, hypotaurine, and β-alanine.

One of the first studies showing the ability of carnosine to activate the Nrf2 pathway was carried out by Alsheblak et al. [249]. By employing a rat model of carbon tetrachloride (CCl_4_)-induced hepatic injury, the authors demonstrated the ability of carnosine (250 mg/kg), administered through i.p. injection daily for a total of six weeks, to rescue the nuclear Nrf2 levels in rat livers that were down-regulated in the presence of CCl_4_ only. As expected, based on hundreds of genes under its control, the positive modulation of Nrf2 nuclear translocation by carnosine was linked to enhanced antioxidant and anti-inflammatory activities [249]. It is well-known that there is a strong link between T2DM and CVD, representing the most prevalent cause of morbidity and mortality in diabetic patients [250]. In a disease model that was represented by streptozotocin induced diabetic rats, carnosine treatment improved the rats’ learning and memory disturbances through the positive modulation of the NF-κB/Nrf2/HO-1 pathway that was paralleled by the attenuation of astrogliosis, a reduction in hippocampal acetylcholinesterase activity, and the amelioration of oxidative stress (decreased lipid peroxidation and increased SOD activity and GSH levels) and neuroinflammation (decreased NF-κB and TNF-α) [251]. The protection exerted by carnosine through the modulation of Nrf2 could also explain the ability of this dipeptide to counteract the cardiotoxicity that was induced by DOX in rabbits [252].

Moving to preclinical in vitro studies, Zhao et al. have demonstrated that carnosine is able to protect mouse podocytes against glucose-induced apoptosis, with a key role played by the activation of both the PI3K/Akt and Nrf2 pathways, culminating in the inhibition of ROS production [253]. In a different study employing podocytes that was carried out by Scuto et al., carnosine induced cellular stress tolerance and resilience pathways, including Sirt1 and HO-1, and was highly effective in reducing high-glucose-induced glycative and lipoperoxidative stress [254]. In particular, carnosine was able to dose-dependently reduce the increase in 4-hydroxy-trans-2-nonenal and protein carbonylation that was observed as a consequence of glucose treatment. In a recent work, the modulation of pro-oxidant and pro-inflammatory activities of M1 activated macrophages by carnosine was proven [216]; specifically, carnosine increased the expressions of Nrf2, as well as of its principal down-stream gene HO-1, that was accompanied by the amelioration of cell energy state, a decrease in pro-oxidant enzymes expression and lipid peroxidation, the restoration and/or increase in the intracellular pool of antioxidant enzymes, and a decrease in inflammation. In a different recent study that was carried out by Afifi et al. on the synergistic effect of carnosine and aminoguanidine in counteracting the hepatic encephalopathy (induced by thioacetamide i.p. injection) in rats through the activation of the Nrf2/HO-1 pathway, nitric oxide inhibition was demonstrated [255]. During the last two decades, the antioxidant and anti-inflammatory effects of the carnosine analog polaprezinc, a chelated compound of zinc and L-carnosine, have also been investigated [256]. Interestingly, this compound has also been proposed as an effective natural antidote against *Helicobacter pylori* infection, considered to be a worldwide problem with an increasing burden on the health sector because of its increasing rate of resistance.

A very recent randomized controlled study that was carried out by Ibrahim and collaborators demonstrated that 10 days of modified bismuth quadruple therapy fortified with polaprezinc (also known as zinc carnosine) was more effective than 14 days of conventional triple therapy in eradicating *H. pylori* infection, without additional significant adverse events [257]. Of note, a different recent study employing polaprezinc-loaded antioxidant electrospun membrane demonstrated the ability of this formulation to up-regulate the Nrf2/HO-1/SOD-1 signaling molecules in a concentration-dependent manner [258].

A clinically relevant example of the use of carnosine as a potential antidote against drug-induced toxicity has recently been given by Yehia et al. [259]. Oxaliplatin is a chemotherapic agent that is known to induce acute neurotoxicity. A total of 65 patients were recruited in this pilot study, 31 of which received FOLFOX-6 (oxaliplatin and leucovorin), while 34 patients received FOLFOX-6 and daily oral L-carnosine (500 mg) throughout the treatment period (three months). While the treatment of colorectal cancer patients with FOLFOX-6 led to a significant down-regulation of Nrf2, thus making patients more susceptible to oxidative stress and inflammation, the oral co-administration of carnosine exerted a neuroprotective effect against oxaliplatin-induced peripheral neuropathy by targeting the Nrf-2 (up-regulation) and NF-κB (down-regulation) pathways. In particular, this clinical study demonstrated for the first time the high therapeutic potential of L-carnosine as an antidote, and a strong impact of this peptide on oxidative stress (Nrf-2 and malondialdehyde (MDA)) and inflammatory (NF-κB and TNF-α) and apoptotic (caspase-3) markers in patients with colorectal cancer. The high clinical relevance of this study stems from the evidence that L-carnosine protected 40% of patients from peripheral neuropathy, an effect that is paralleled by an elevation of Nrf-2 mean serum levels (by 38.7%) in combination with a reduction in MDA levels (−51.8%), suggesting that the activation of Nrf2 with carnosine might represent a novel pharmacological strategy to prevent drug-induced neurotoxicity.

## 6. Conclusions

Different drug classes such as antineoplastic drugs (anthracyclines, cyclophosphamide, 5-fluorouracil, taxanes, tyrosine kinase inhibitors), antiretroviral drugs, and antipsychotic and immunosuppressant drugs are known to induce cardiotoxic and neurotoxic effects. The onset of drug-induced cardiotoxicity and neurotoxicity represents a clinically relevant challenge in the field of clinical toxicology and the identification of the molecular mechanisms underlying these adverse drug effects represent an essential step in developing innovative antidotes. Unfortunately, dexrazoxane is the only recently approved drug that is able to act as an antidote in this field, preventing DOX-related cardiotoxicity, but new antidotes are needed to suppress oxidative stress and counteract both drug-induced cardiotoxicity and neurotoxicity. In the present preview, we have provided evidence that the negative modulation of the Nrf2 pathway is a mandatory step in the pathogenesis of drug-induced cardiotoxicity and neurotoxicity, whereas an activation of this pathway sustains the endogenous antioxidant machinery protecting cardiomyocytes and neurons from oxidative stress and related inflammatory phenomena through the enhanced expression of cytoprotective enzymes such as SOD, HO-1, glutathione peroxidase 2, and GSTs. The Nrf2 pathway might therefore be considered as a novel pharmacological target in the field of clinical toxicology to develop innovative antidotes and counteract drug-induced cardiotoxicity and neurotoxicity.

In this new scenario, carnosine, an endogenous dipeptide that is characterized by strong antioxidant, anti-inflammatory, and neuroprotective properties, can play a novel and interesting role. Numerous preclinical and clinical studies have been published on the therapeutic potential of carnosine dipeptide. Despite this, as underlined in the present review, something is still missing or needs improvement, specifically in the context of clinical studies in which a high clinical and methodological heterogeneity has been observed. Future clinical studies should adopt a common methodology in terms of peptide administration and study design in order to better understand the clinical efficacy of carnosine. Nevertheless, when considering the strong preclinical evidence on the ability of carnosine to rescue/activate the Nrf2 pathway, we examined the therapeutic potential of this endogenous peptide as a novel antidote that is able to rescue the Nrf2 pathway, hypothesizing, for the first time, that this peptide can be proposed in future clinical studies to prevent drug-induced cardiotoxicity and neurotoxicity.

## Data Availability

No applicable.
